# Editorial: Community series in adaptor molecules in T cell signaling, volume II

**DOI:** 10.3389/fimmu.2023.1243039

**Published:** 2023-07-26

**Authors:** Sutatip Pongcharoen, Anne Spurkland, Cosima T. Baldari

**Affiliations:** ^1^Division of Immunology, Department of Medicine, Faculty of Medicine, Naresuan University, Phitsanulok, Thailand; ^2^Department of Molecular Medicine, Institute of Basal Medical Sciences, University of Oslo, Oslo, Norway; ^3^Department of Life Sciences, University of Siena, Siena, Italy

**Keywords:** T cell, adaptor molecules, TCR signaling, signal transduction, T cell receptor

T cells are the essential effectors of the adaptive immunity. They use their unique receptor, the T cell receptor (TCR), to specifically recognize and respond to antigens associated with the major histocompatibility complex (MHC) molecules. Signal transduction of the TCR is initiated with the function of the CD3 subunits of the TCR complex. The very first event in TCR/CD3 complex activation is the phosphorylation of the cytoplasmic tails of CD3ζ by the tyrosine kinase Lck, allowing the recruitment of the kinase ZAP70 to phosphorylate a number of downstream mediators and effectors to amplify TCR/CD3 signaling.

Adaptor molecules couple the TCR/CD3 complex to a variety of signaling molecules involved in T cell activation and as such play a key role as signal amplifiers. Adaptors include both transmembrane and cytosolic molecules, such as LAT, SLP-76, GRB2, SHC, and Gads. While some adaptor molecules, like TSAd and Nck, may participate in proximal TCR/CD3 signaling by recruiting Lck, other adaptors, such as PAG and SIT, may negatively regulate TCR signaling, thereby contributing to signal termination. Signaling by the TCR has been heavily investigated for exploitation in cancer immunotherapy through approaches involving CAR T cells or other engineered T cells. Adaptor proteins have fundamental roles in the fine-tuning the tightly controlled intricate process of TCR/CD3 signaling both in T cell homeostasis and immune responses. Harnessing their signal modulating properties may help develop better cancer immunotherapies. Additionally, adaptor molecules are among the components of the TCR signaling cascade being investigated for their implication in pathological conditions including autoimmunity and hematologic malignancies, and some adaptor molecules have been identified as therapeutic targets to treat or alleviate those conditions. This Research Topic focuses on adaptor proteins that participate in T cell signaling and thus modulate the adaptive immune response.

Luff et al. (1) have documented the indispensable role of phosphoinositide 3-kinase δ (PI3Kδ) that is recruited to and activated at the TCR signalosome. Using mass spectrometry, biochemical approaches and CRIPR gene editing, they demonstrated that PI3Kδ interacts with many adaptor proteins involved in naïve T cell activation and proliferation. Importantly, these novel interactions between PI3Kδ and adaptor proteins occurring in T cells may be applied to control the threshold of activation and diversify the inputs for PI3K signaling in effector T cells.

Gartshteyn et al. (2) have reviewed the function of the signaling lymphocyte activation molecules (SLAM), a family of T cell transmembrane co-receptors that modulate T cell response to antigens. SLAM signal via interaction with the adaptor protein SH2D1A or SLAM associated protein (SAP). The latter functions to recruit tyrosine kinases and shield phosphorylated sites from dephosphorylation. They suggested that a balanced SLAM-SAP signaling in T cells is required for healthy immunity and that alteration of this pathway could be associated with autoimmune disorders. Hence, SLAM-SAP signaling could be a promising treatment target for autoimmunity.

Lo and Weiss (3) have revisited to the critical role of the linker for activation of T cells (LAT) in T cell receptor ligand discrimination. Self- and non-self ligand discrimination has been believed to be a core principle underlying T cell-mediated immunity. The authors discussed new evidence that supports the kinetic proofreading model of TCR ligand discrimination that is important for the outcome of TCR signaling. The role of LAT and the SH2 domain-containing leukocyte protein of 76 KDa (SLP-76) in thymocyte development and activation of mature T cells is reviewed by Dinur-Schejter et al. (4) Besides clear evidence from several murine models, the defects in LAT also cause human inborn errors of immunity. Similarly, the function of SLP-76 has been shown to be essential in T cell lines, mouse models and a human infant reported with autoimmune and lymphoproliferative manifestation. Thus, these two adaptor molecules are important for normal immunity. In addition, the role of LAT as a possible checkpoint for T cell development and activation has been demonstrated by Arbulo-Echevaria et al. (5) They showed that mice expressing mutant LAT (glycine to aspartic acid at position 135) have an increase in the percentage of CD4^+^CD8^+^ double-positive but a decrease in that of CD4^+^ and CD8^+^ single-positive thymocytes. However, in the periphery these mice have reduced a percentage of CD8^+^ T cells and increased γδT cells. Additionally, Ruminski et al. (6), using CRISPR gene editing and affinity purification coupled with mass spectrometry, have revealed the important role of GRB2 family adaptors in TCR signaling plasticity relating to the SLP-76 interactome.

Borroto et al. (7) have extended these essential role of the proline-rich sequence in CD3ε that binds to Nck adaptor protein in the differentiation and function of pro-inflammatory Thγδ17 cells implicated in an imiquimod model of skin inflammation. This mouse model resembles psoriasis in humans, hence suggesting the novel approach for the treatment of this disease.

The adhesion and degranulation-promoting adaptor protein (ADAP) and the Src kinase associated protein 1 and 2 (SKAP1 and SKAP2) have been reviewed by Dadwal et al. (8) for their important and multiple roles in TCR/CD3 signalosome and signal transduction in T cells. The authors have pointed out the crucial roles of these molecules relating to the function of leucocyte function associated antigen 1 (LFA-1), LAT and SLP-76. In mice, defects in ADAP translate into inflammatory processes leading to demyelination and axonal damage. The authors suggested further investigation on T cells with ADAP mutations in humans with small-platelet thrombocytopenia and increased bleeding tendency. In their review, Liu et al. (9), expanded on this topic and explained how SKAP1 regulates the activation of integrin and optimizes the cell cycling of proliferating T cells through multiple interactions with various mediators.

Velnati et al. (10) have reported a novel signaling pathway mediated by binding of Wiskott-Aldrich syndrome protein (WASp) and diacylglycerol kinase alpha (DGKα). Efficient TCR signaling is known to require inhibition of DGKα triggered in response to SAP activation. The authors showed that non-catalytic region of tyrosine kinase-1 (Nck-1) and the small G protein CDC42 connect the interaction of WASp and DGKα leading to the inhibition of DGKα activity and T cell IL-2 response.

Signaling transduction in T cells eventually leads to the NF-κB transcription factor activation. This process is a critical step for T cell survival, proliferation, differentiation and function. O’Neill et al. have demonstrated the existence of a signalosome containing three interacting proteins, caspase recruitment domain family member 11 (CARD11), B-cell lymphoma/leukemia 10 (BCL10), and mucosa-associated lymphoid tissue 1 (MALT1), called the CBM complex. MALT1 is known to be a scaffold for the E3 ligase tumour necrosis factor (TNF) receptor-associated factor 6 (TRAF6). The authors showed in a mouse model that the CMB is crucial for balancing T cell activation and homeostasis. In addition, Oikawa et al. (11) have added the new finding that this CBM complex is ubiquitinated by the E3 ubiquitin ligase called LUBAC. Using Jurkat cells in various assays and mathematical simulation, they demonstrated that LUBAC controls the activation of NF-κB, critically mediating T cell functions. In addition, Hornick and Bishop have reviewed the role of TRAF3 as a mediator of T cell differentiation and effector function. The essential function of TRAF3 has been demonstrated both in murine models and human disease caused by germline TRAF3 deficiency. The affected patients have autoimmunity, immunodeficiency, and a predisposition to B cell malignancies.

Collectively, this Research Topic contributes to provide new insights into T cell biology area involving adaptor proteins, including proximal and distal TCR signaling, thymocyte development and T cell differentiation, signalosomes relating to T cell signal transduction and regulation of cytokines and other signaling mediators. The knowledge obtained can set the foundation to novel approaches for treatment of autoimmunity and cancer.

## Author contributions

SP, AS, and CB drafted and edited the present editorial. All authors contributed to the article and approved the submitted version.
